# Evaluating zero‐shot prediction of monomeric protein design success by AlphaFold, ESMFold, and ProteinMPNN

**DOI:** 10.1002/pro.70453

**Published:** 2026-01-20

**Authors:** Mario Garcia, Sugyan M. Dixit, Gabriel J. Rocklin

**Affiliations:** ^1^ Department of Pharmacology & Center for Synthetic Biology Northwestern University Feinberg School of Medicine Chicago Illinois USA; ^2^ Robert H. Lurie Comprehensive Cancer Center Northwestern University Feinberg School of Medicine Chicago Illinois USA

**Keywords:** de novo protein design, protein biophysics, sequence prediction, structure prediction

## Abstract

De novo protein design has enabled the creation of proteins with diverse functionalities that are not found in nature. Despite recent advances, experimental success rates remain inconsistent and context‐dependent, posing a bottleneck for broader applications of de novo design. To overcome this, structure and sequence prediction models have been applied to assess design quality prior to experimental testing to save time and resources. In this study, we examined the extent to which AlphaFold, Protein MPNN, and ESMFold can discriminate between experimentally successful and unsuccessful designs. We first curated a benchmark dataset of 614 experimentally characterized de novo designed monomers from 11 different design studies between 2012 and 2021. All predictive models demonstrated moderate ability to discriminate experimental successes (expressed, soluble, monomeric, and fold with the correct secondary structure) from failures. Still, many failed designs have better confidence metrics than successful designs, and confidence metrics were topology‐dependent. Among all computational models evaluated, ESMFold average predicted local‐distance difference test (pLDDT) yielded the best individual performance at distinguishing between successful and unsuccessful designs. A logistic regression model combining all confidence metrics provided only modest improvement over ESMFold pLDDT alone. Overall, these results show that these models can serve as an initial filtering strategy prior to experimental validation; however, their utility at accurately predicting experimentally successful designs remains limited without task‐specific training.

## 1 INTRODUCTION

De novo protein design permits the exploration of protein sequence space beyond the constraints imposed by natural evolution (Huang et al. 2016a). This approach allows for the design of novel protein folds with precise control over the designed structure to achieve specific functions, representing promise for developing novel proteins with applications in medicine and biotechnology (Korendovych and DeGrado 2020). Despite recent breakthroughs in AI‐guided structure prediction and design strategies, experimental success rates of designs remain inconsistent, with rates varying based on the complexity of the designed topology and design objective (Pan and Kortemme 2021). For example, α‐helical protein designs tend to have higher experimental success rates compared to designs rich in β‐sheet content or designs attempting to incorporate functionality (Kortemme 2024; Pan and Kortemme 2021). These inconsistencies emphasize the need for improved computational models to better predict which designs will likely succeed experimentally.

Protein design can fail at many experimental stages, including through poor expression, low solubility, aggregation, folding to an undesired or non‐functional conformation, or low overall folding stability. Implementing computational strategies to filter out bad designs prior to characterization can reduce the experimental resources and cost associated with validating designs (Hermosilla et al. 2024; Peñas‐Utrilla and Marcos 2022).

Over the last several years, deep learning models have increasingly been applied for filtering computational designs. Structure‐based prediction models such as AlphaFold, inverse folding prediction models such as Protein MPNN, and protein language models such as ESMFold have been integrated in design strategies as final validation steps to remove poor designs from experimental characterization (Abramson et al. 2024; Dauparas et al. 2022; Jumper et al. 2021; Lin et al. 2023; Sumida et al. 2024; Wang et al. 2022; Watson et al. 2023). Although these computational models have shown great capabilities at sequence and structure prediction, they were not trained to predict protein biophysical properties such as expression, aggregation, or stability. In AlphaFold predictions, regions with low pLDDT scores often correspond to intrinsically disordered segments lacking a stable three‐dimensional fold (Ruff and Pappu 2021). However, studies have shown that AlphaFold pLDDT provides little discrimination between experimentally stable and unstable designs (Liu et al. 2022; Kim et al. 2022). As a result, many de novo designed proteins achieve highly confident model metrics even when these designs fail to express or fold under typical experimental conditions (Chu et al. 2024; Frank et al. 2024; Ruff and Pappu 2021; Wicky et al. 2022). This highlights the importance of assessing the performance of sequence and structure prediction models when tasked to discriminate between experimental successes and failures in de novo design. Furthermore, design and prediction are separate challenges. Even if a protein design method arose with near‐perfect success at a certain design task, it would remain important to accurately predict whether diverse sequences generated by other approaches could also achieve the design objective.

In this study, we aim to quantify the extent to which existing deep learning models developed for sequence and structure prediction can distinguish between experimentally successful and unsuccessful de novo protein designs. To accomplish this, we compiled a dataset of 614 experimentally characterized de novo designed proteins covering many different designed topologies from the past decade. All proteins were designed using Rosetta‐based methods to fold into soluble monomers without deep learning, indicating that these designs all had strongly favorable energies according to a classical protein design energy function (Alford et al. 2017). Since these models have never been trained on experimental outcomes such as protein expression, solubility, or folding stability, asking them to predict design success is a “zero‐shot” evaluation of these models. This analysis provides a baseline for their performance in predicting design success. We also use our benchmark dataset containing many unique folds to examine the influence of protein topology on model confidence metric performance and accuracy.

## 2 RESULTS

### 2.1 Roughly half of the designs across 11 studies met all experimental success criteria

**FIGURE 1:**
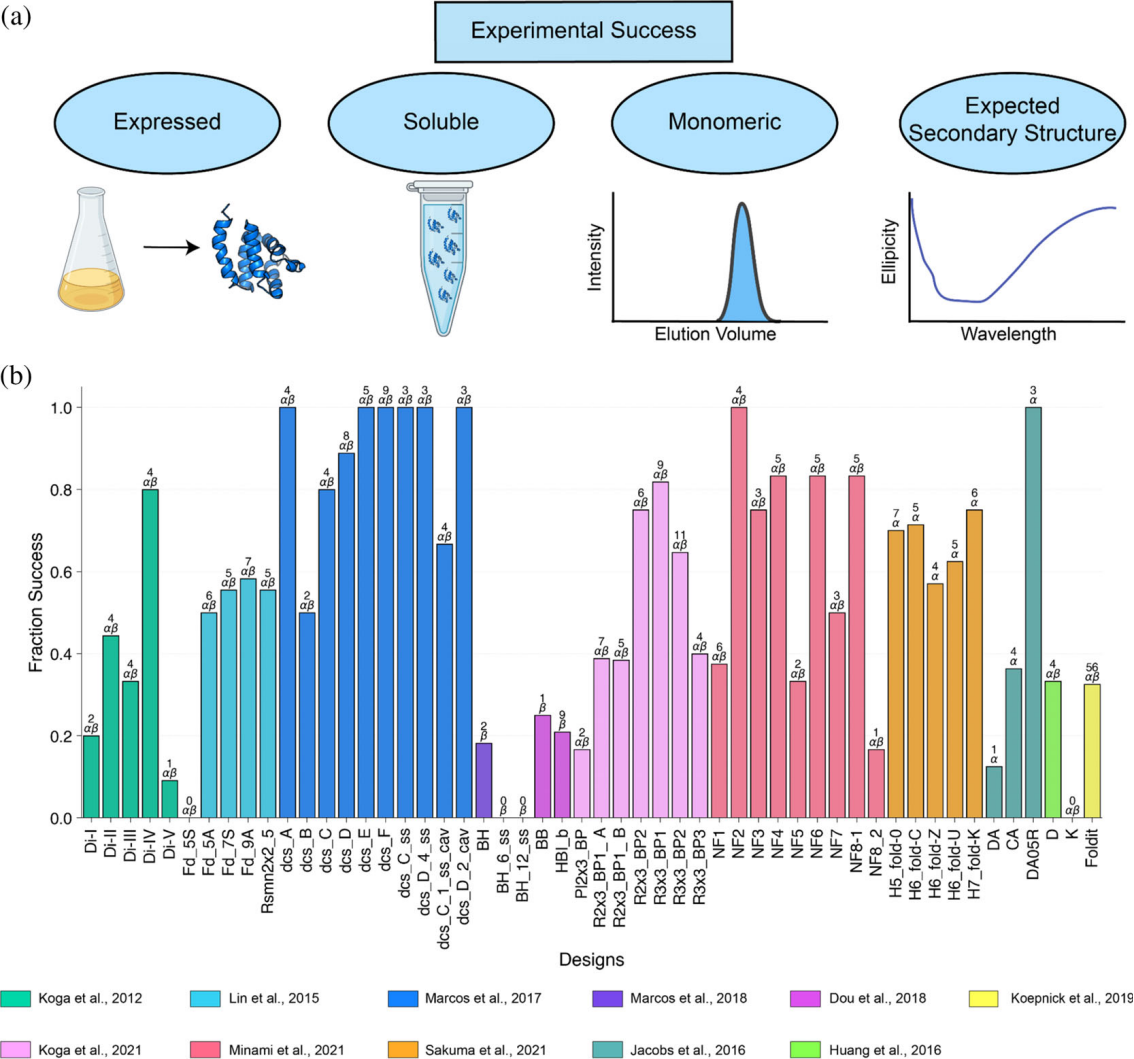
Experimental success rates vary depending on design objective and topology. (a) Schematic depiction of criteria used to define experimentally successful de novo designs in this analysis. A design was considered successful if it was expressed, soluble, monomeric, and folded into the intended secondary structure. (b) Fraction of experimentally successful designs for each design topology. Design topologies consisted of all α‐helical proteins (α), all β‐sheet proteins (β), or mixed α/β proteins (α/β). The number of successful designs within each topology is reported above each bar. Bars for each design topology are colored according to the reported article.

**FIGURE 2:**
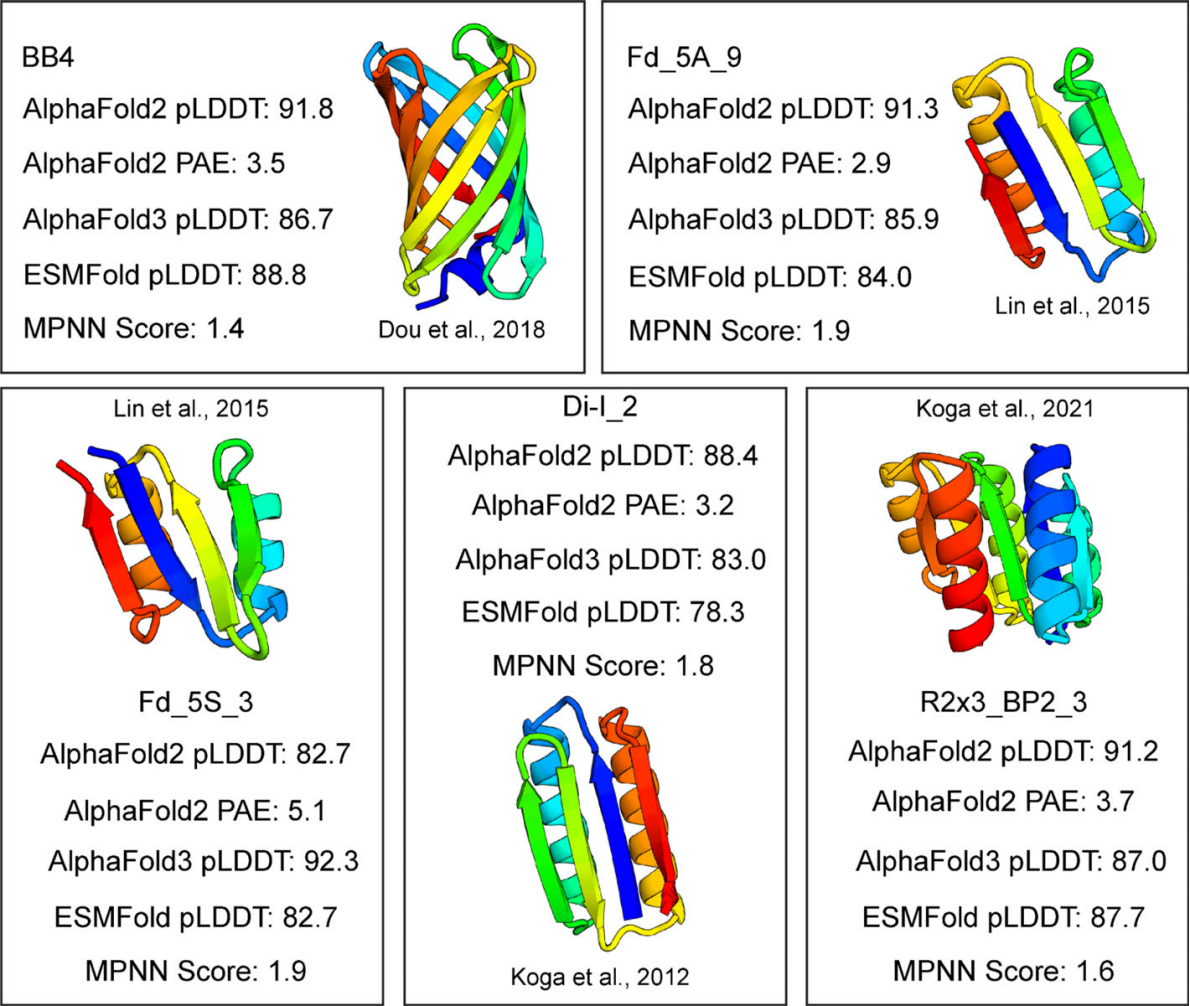
Soluble designs that failed to fold into intended secondary structures despite high AlphaFold confidence. High confidence (pLDDT >82) AlphaFold predicted structure of designs that were experimentally soluble but failed to fold into intended secondary structures, assessed by circular dichroism.

We compiled 11 de novo protein design studies that collectively included 614 individual monomeric de novo designs. Each study typically focused on several different design “topologies” specifying the lengths and connectivity of secondary structural elements in each design. Overall, 269/614 (43%) were experimentally successful (expressed, soluble, monomeric, and with the correct secondary structure). Success rates varied from study to study and topology to topology (Figure 1b). For example, whereas most (88%) of the curved β‐sheet proteins in Marcos et al. (Marcos et al. 2017) met all experimental success criteria, a much smaller fraction (21%) of the β‐barrel designs from Dou et al. (Dou et al. 2018) were experimentally successful. Many of the lowest success rates came from efforts to design β‐sheet rich proteins, such as β‐barrel and jellyroll designs (Dou et al. 2018; Marcos et al. 2018). Only 16% of failed designs were due to poor expression, while the majority of designs failed because of insolubility and aggregation (65% of failures) (Figures S1 and S2, Supporting Information). Still, many soluble, monomeric designs failed to show the intended secondary structure via circular dichroism (59/345). Figure 2 displays examples of soluble designs with high‐confidence predicted structures from AlphaFold2 but that failed to adopt the designed secondary structure. These designs indicate that soluble, monomeric expression is not sufficient to establish proper folding under the experimental conditions tested (typically room temperature and neutral pH). Of course, these proteins may still form their predicted structures under alternative experimental conditions, such as lower temperature and/or the presence of chemical stabilizers. Although the full dataset is made available in Supporting Information, we removed 44 designs with solved NMR structures for further analysis to ensure unbiased AlphaFold performance.

### 2.2 Structure prediction confidence metrics moderately discriminate between good and bad designs

**FIGURE 3:**
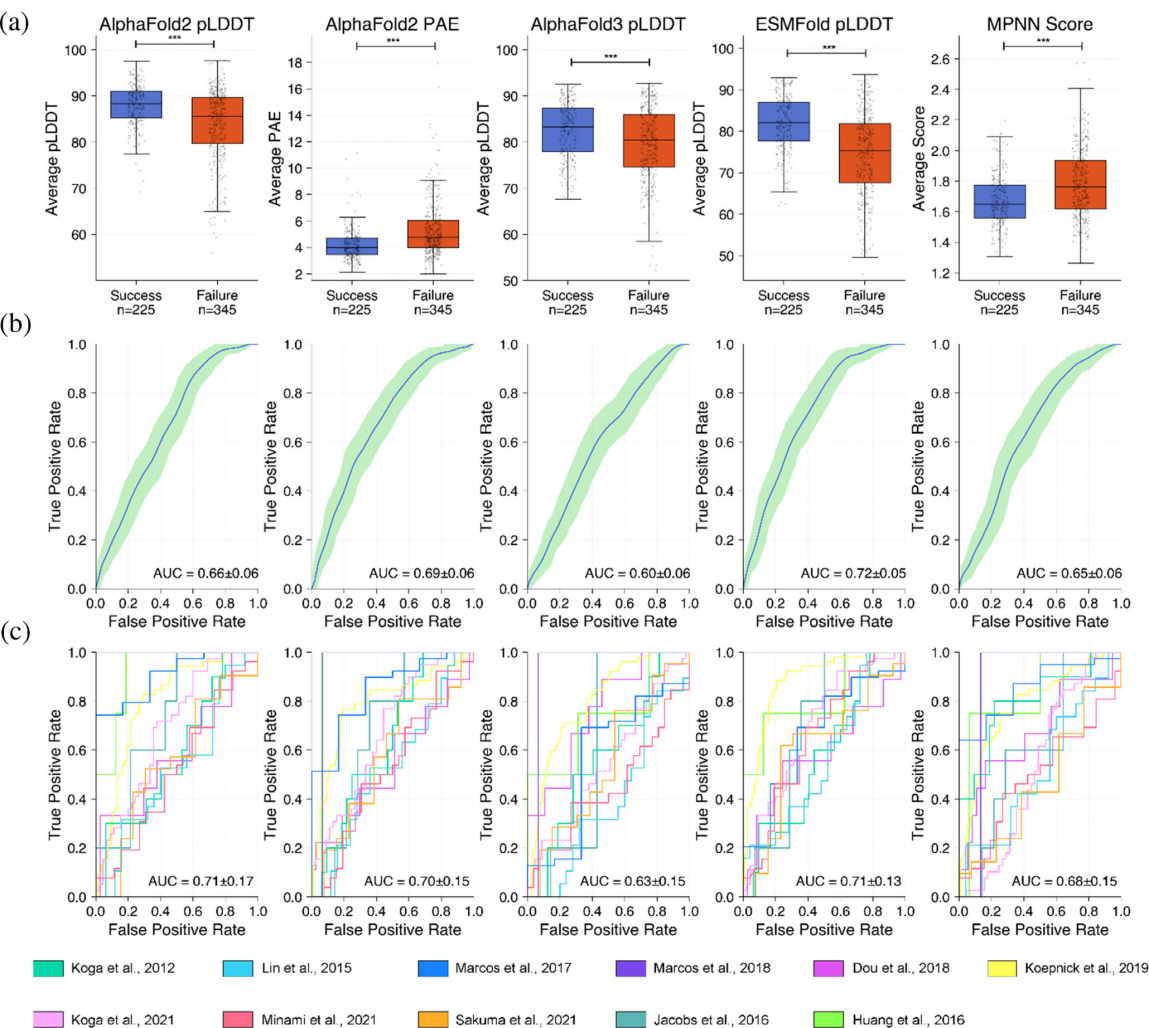
ESMFold pLDDT provides the greatest discrimination between experimentally successful and unsuccessful designs. (a) Boxplots displaying distributions of confidence metrics between experimentally successful (blue) and unsuccessful (orange) designs with successful designs displaying better confidence metrics on average. Asterisks indicate statistical significance based on a Mann–Whitney *U* test (***p < 0.001). (b) ROC curves for each confidence metric displaying the average AUC values. The shaded region represents the 95% confidence interval computed from bootstrapping. (c) ROC curves for each confidence metric computed using leave‐one‐fold‐out cross‐validation. Reported AUC values correspond to the mean ± standard deviation across studies.

Overall, experimentally successful designs had better confidence metric distributions compared to unsuccessful designs across all computational models (Figure 3a). Still, discrimination was far from perfect, with Receiver Operating Characteristic (ROC) curve area under the curve (AUC) scores ranging from 0.60–0.72 for the different confidence metrics (Figure 3b). ESMFold's average pLDDT had the highest AUCs out of the different confidence metrics (0.72 ± 0.05, mean ± SD from bootstrapping), whereas AlphaFold3 pLDDT had the lowest AUC (0.60 ± 0.06, mean ± SD from bootstrapping). Confidence metric performance also varied across the different design studies (which focused on different design goals), further illustrating the difficulty of establishing thresholds that are generalizable across different design contexts. Still, confidence metric performance within individual design studies showed similar trends as when examining all studies together, with AUCs ranging from 0.63 to 0.71 for the different confidence metrics (Figure 3c). ESMFold and AlphaFold2 average pLDDT had the highest average AUCs across articles (0.71 ± 0.13 and 0.71 ± 0.17, mean ± SD across individual articles for ESMFold and AlphaFold2).

**FIGURE 4:**
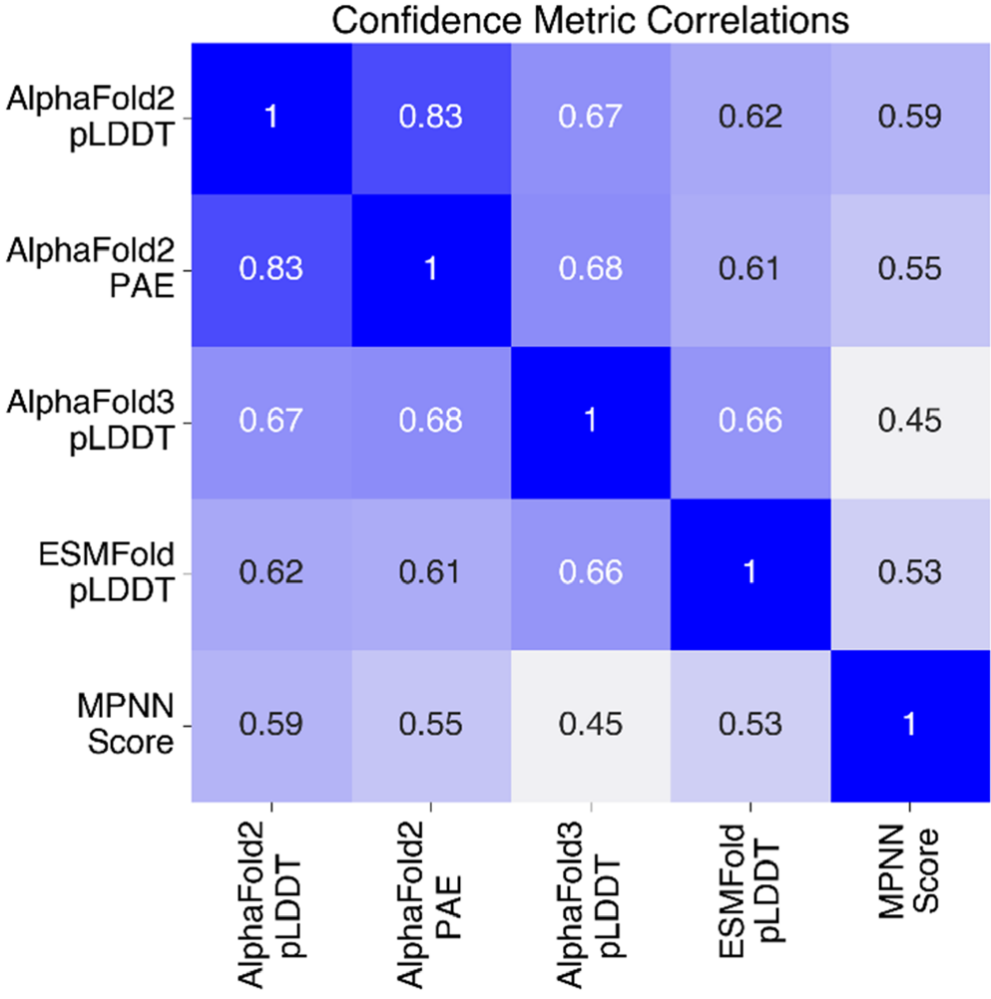
Confidence metrics were moderately correlated. Spearman correlation matrix showing absolute correlation between different confidence metrics.

**FIGURE 5:**
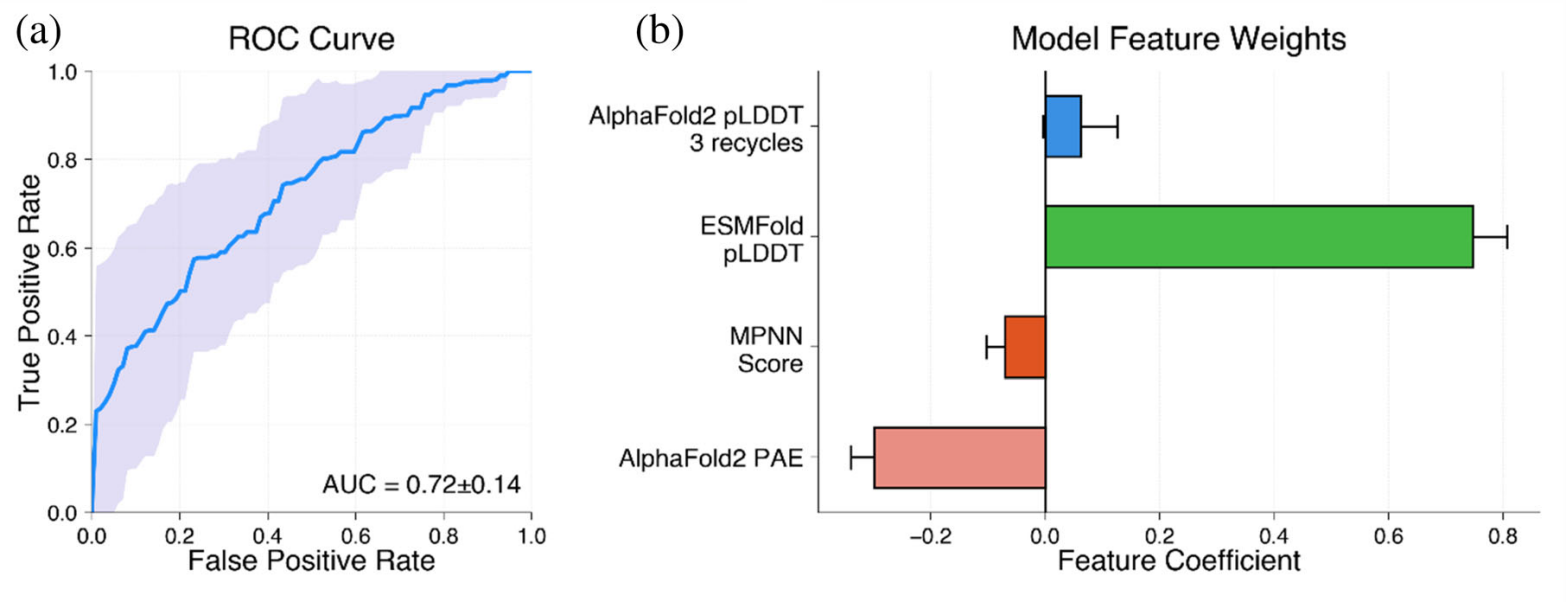
Combining confidence metrics into a logistic regression model shows no significant improvement in average AUC. (a) ROC curve for logistic regression model trained using all confidence metrics to predict experimental success. The ROC curve displays the mean (dark blue) and standard deviation across studies evaluated using a leave‐one‐fold‐out cross‐validation. (b) Feature weights from the logistic regression model indicating the relative contribution of each metric to the model. ESMFold pLDDT showed the highest feature weight.

AlphaFold2 pLDDT values generated with different numbers of recycles were highly correlated (Spearman = 0.94) and showed no differences in AUC (Figure S3). A total of 31/570 designs showed an increase in pLDDT >5 using 25 recycles, with three designs increasing their pLDDT by more than 15 points. These 31 designs were primarily FoldIt designs (23/31) from Koepnick et al. (Figure S3). Furthermore, different confidence metrics captured different information, with Spearman correlations between metrics ranging from 0.45 to 0.83 (Figure 4). Still, despite differences between these methods, we found that combining these metrics using a logistic regression model (ROC AUC of 0.72 ± 0.14, mean ± SD from held out test articles) showed no significant improvement over the best individual methods (Figures 5 and S7). Combined metric models also showed limited ability to predict whether designs would be expressed, soluble, monomeric, and fold into their intended secondary structure (Figure S8).

### 2.3 Confidence metrics were not generalizable across design topologies, preventing a reliable threshold from being established for experimental success

**FIGURE 6:**
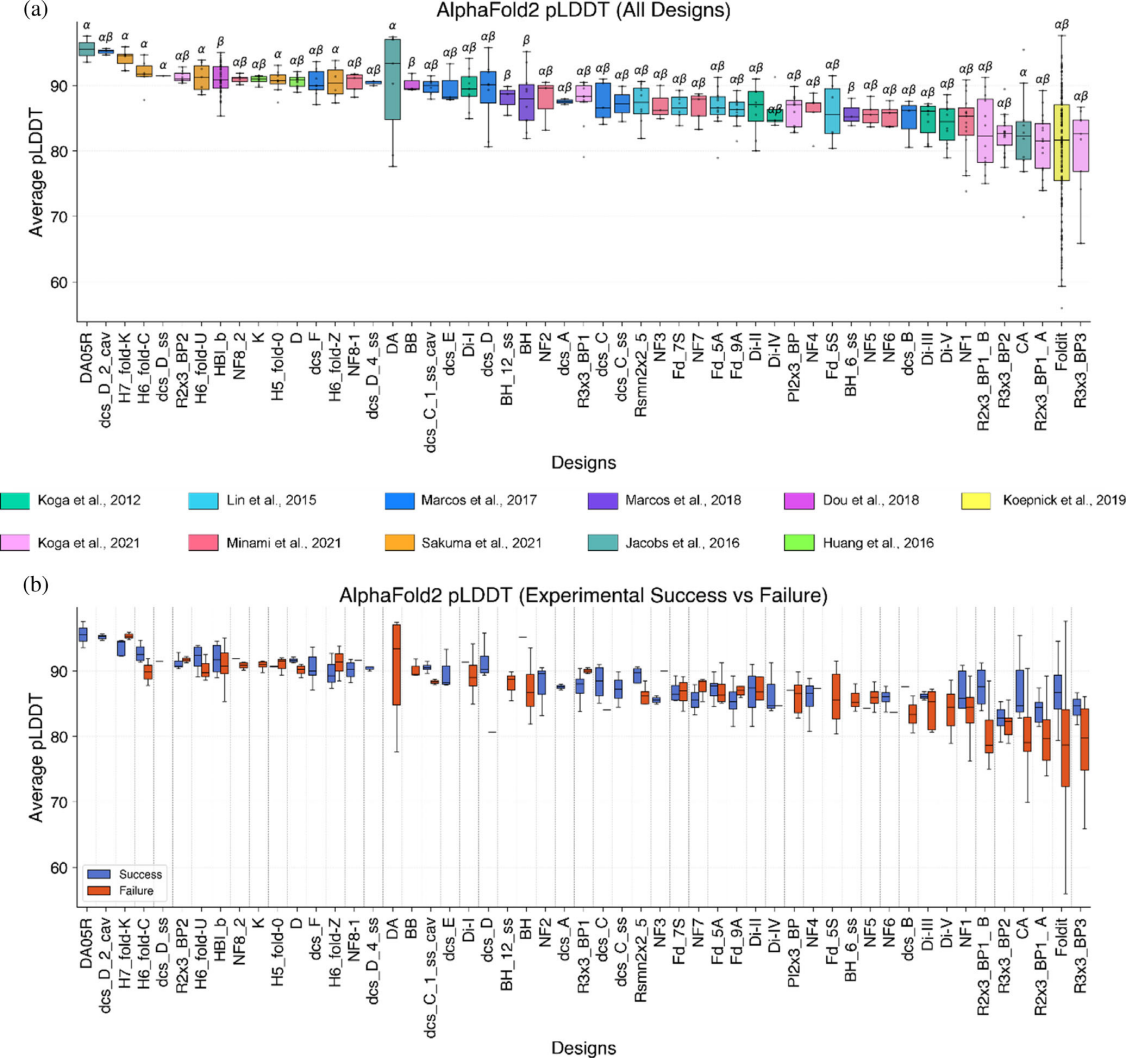
AlphaFold2 average pLDDT distributions vary across different design topologies. (a) Distribution of AlphaFold2 average pLDDTs for designs included in this analysis. (b) Distribution of AlphaFold2 average pLDDTs comparing experimentally successful designs (blue) and unsuccessful designs (orange) for each design topology.

We then analyzed confidence metrics across different design topologies, focusing initially on AlphaFold2 average pLDDT values (Figures 6 and S4–S6). Importantly, we observed that the distribution of AlphaFold2 pLDDT values varied across different design topologies (Figure 6a). This topology‐dependence indicates that there is no clear “threshold” in AlphaFold2 pLDDT (or ESMFold pLDDT; Figure S5) for experimental success. Failed designs from “easily predictable” topologies on the left of Figure 6 often had higher pLDDT scores than successful designs from “difficult to predict” topologies on the right of Figure 6. Although a clear threshold was not established, filtering designs to select the top 50% of designs based on AlphaFold2 and ESMFold average pLDDT increased the design success rate from 39% to 57% within the filtered subset (Figure S9). While AlphaFold2 (and ESMFold) pLDDT scores remain useful ranking metrics within an individual topology, this illustrates the difficulty of comparing designs between topologies and using pLDDT as a proxy for experimental success.

## 3 DISCUSSION

In this study, we found that sequence and structure prediction models offer a moderate ability to distinguish between experimentally successful and unsuccessful de novo designs, with ESMFold average pLDDT providing the highest discrimination. Integrating confidence metrics from sequence and structure prediction models into a logistic regression model did not result in significant improvement compared to ESMFold pLDDT alone. Notably, many unsuccessful designs received better confidence metrics than successful designs across all models, and confidence metrics were correlated with the design topology. Examining the predicted structures of these models can potentially overcome these inconsistencies. For this analysis, we did not have access to the designed backbones; however, root mean square deviation (RMSD) calculations between predicted backbone structures and the intended backbone designs can be implemented in future work to identify large deviations from the intended backbone design to further filter poor designs (Hermosilla et al. 2024). Filtering designs based on RMSD calculations can improve experimental success designs (Liu et al. 2022; Sumida et al. 2024). Moreover, computational models are continuously being developed to predict experimental properties such as solubility, aggregation, conformational dynamics, and stability (Dieckhaus et al. 2024; Ferrari et al. 2025; Li and Ming 2024; Martell et al. 2025; Monteiro da Silva et al. 2024). Incorporating these models into design strategies could further improve the experimental success rates. As large‐scale experimental datasets become available, we anticipate that training and fine‐tuning these models on this data will help improve the inconsistencies in experimental success rates of de novo designs.

## 4 METHODS

### 4.1 Dataset construction

The design studies selected for this analysis focused on the de novo design of monomeric proteins (Dou et al. 2018; Huang et al. 2016b; Jacobs et al. 2016; Koepnick et al. 2019; Koga et al. 2012; Koga et al. 2021; Lin et al. 2015; Marcos et al. 2017; Marcos et al. 2018; Minami et al. 2023; Sakuma et al. 2024). These studies did not include AlphaFold, Protein MPNN, or ESMFold during the design process. Forty‐four designs contained NMR structures that were deposited in the RCSB Protein Data Bank and were excluded from analysis of confidence metric performance. All monomeric designs in the dataset were experimentally characterized using expression in *E. coli*, protein purification, size exclusion chromatography combined with multi‐angle light scattering (SEC‐MALS), and secondary structure assessment by circular dichroism (CD). We defined a design to be experimentally successful if it was expressed, soluble, monomeric, and had a CD spectrum consistent with the intended design fold as reported in each study (Figure 1a). Protein designs that did not report experimental success or designs needing to be stabilized as dimers were excluded from the final analysis.

### 4.2 AlphaFold modeling

AlphaFold2 (AlphaFold 2.1.1 version) and AlphaFold3 (AlphaFold 3.0.0 version) were used to generate predicted structures for all monomeric de novo designs in the dataset (Abramson et al. 2024; Jumper et al. 2021). For each sequence, five predicted protein structures were generated using the AlphaFold2 monomer model. The confidence for each structure was determined by the pLDDT metric. The structure with the highest average pLDDT value was selected as the best‐ranked AlphaFold predicted structure. AlphaFold2 predicted structures were generated using both the default protocol of 3 recycles and an extended protocol with 25 recycle steps (Jumper et al. 2021). In addition, the AlphaFold2 PTM model was used to compute the average predicted aligned error (PAE) of each protein design. The average AlphaFold pLDDT and PAE of each design were used as confidence metrics to assess model performance when predicting experimental success. A higher AlphaFold pLDDT and a lower PAE indicate better model predictive performance.

### 4.3 ProteinMPNN modeling

We used the highest‐ranked AlphaFold predicted structures with the default recycle parameter as the input backbone for Protein MPNN (Dauparas et al. 2022). The computed Protein MPNN score (average negative log probability of each amino acid in the designed sequence) for the original sequence was used to assess the model's performance in predicting experimental success, with a lower Protein MPNN score indicating better performance.

### 4.4 ESMFold modeling

To assess the ability of protein language models to predict experimental success, ESMFold was applied to all de novo designed proteins in the dataset (Lin et al. 2023). ESMFold (esmfold_v1) was used to predict the structures for all designs. ESMFold performance was assessed using the average pLDDT of the predicted structures. A higher average pLDDT indicates better model performance for ESMFold.

### 4.5 Statistical analysis

We assessed the ability of each confidence metric to discriminate between experimentally successful designs from failures using a two‐sided Mann–Whitney *U* test.

### 4.6 Logistic regression modeling

ROC curves and regression models were computed using scikit‐learn (Pedregosa et al. 2011). ROC curves for each confidence metric were generated using the full dataset by bootstrapping (sampling 1000 times with replacement). ROC curves for each confidence metric were also generated for each article. Logistic regression models were trained to predict whether designs were expressed, soluble, monomeric, folded into the designed secondary structure, and achieved overall experimental success. Model performance was evaluated using leave‐one‐out cross‐validation, where each design article served as a held‐out test set. For each iteration, the model was trained to predict experimental success using all other articles and tested on a held‐out article. ROC curves were generated for each held‐out article, and the average AUC across held‐out articles was used to assess model performance. Model coefficients were used to evaluate the importance of features.

## AUTHOR CONTRIBUTIONS

**Mario Garcia:** Data curation; methodology; formal analysis; investigation; writing – original draft; visualization. **Sugyan M. Dixit:** Methodology; investigation. **Gabriel J. Rocklin:** Conceptualization; project administration; resources; supervision; funding acquisition; writing – review and editing.

## Supporting information


**Data S1.** Supporting Information.

**Figure S1.** Most de novo designs failed experimentally due to not being monomeric. Counts of experimentally unsuccessful designs for each experimental criteria used to define design experimental success.
**Figure S2.** Distribution of confidence metrics for experimental outcomes of designs. Distribution of confidence metrics comparing successful (blue) and unsuccessful designs (orange) across different experimental outcomes: expression, solubility, monomeric state, and secondary structure.
**Figure S3.** Average AlphaFold2 pLDDT values are highly correlated with different recycle parameters. (a) Scatterplot capturing the relationship between AlphaFold2 average pLDDT for de novo designs computed using different recycle parameters. Green highlighted points indicate designs that increased their average pLDDT by >5 after increasing the recycles, and blue highlighted points are designs that increased their average pLDDT by >15 after increasing recycles. (b) AlphaFold2 predicted structures of designs that increased their average pLDDT >15 with increased recycles. (c) Boxplots showing the distribution of AlphaFold2 average pLDDT values using 25 recycles, comparing experimentally successful (blue) and unsuccessful (orange) designs. The ROC curves for AlphaFold2 average pLDDT (25 recycles) from bootstrapping on the whole dataset and individual article ROC curves.
**Figure S4.** AlphaFold2 average PAE distributions for designed topologies. (a) Distributions of AlphaFold average PAE for designs included in this study. (b) Distribution of AlphaFold average PAE for experimentally successful designs (blue) and unsuccessful designs (orange) for each design topology.
**Figure S5.** ESMFold average pLDDT distributions for designed topologies. (a) Distribution of ESMFold average pLDDT for designs included in this study. (b) Distribution of ESMFold average pLDDT for experimental successful designs (blue) and unsuccessful designs (orange) for each design topology
**Figure S6.** ProteinMPNN score distributions for designed topologies. (a) Distribution of MPNN scores for designs included in this study. (b) Distribution of MPNN score for experimental successful (blue) and unsuccessful designs (orange) for each design topology.
**Figure S7.** Logistic regression models combining all confidence metrics to predict held‐out designs from each study. ROC curves were generated for each held‐out study. The AUC and number of examples (n) are annotated in each plot.
**Figure S8.** Logistic regression models using all confidence metrics trained to predict whether designs were expressed, soluble, monomeric, and folded into designed secondary structure. The ROC curves display the mean (dark blue) and standard deviation across held out test articles.
**Figure S9.** Selecting the top 50% of designs according to AlphaFold2 pLDDT and ESMFold pLDDT increases design success rate to 57%. (a) Scatterplot displaying AlphaFold2 pLDDT and ESMFold pLDDT for all designs. Designs are labeled as experimentally successful (blue) or experimentally unsuccessful (orange). The dashed lines indicate the 50th percentile for each metric and the top‐right quadrant shows the top 50% of designs for both metrics. (b) Counts of experimentally successful and unsuccessful designs for the top 50% designs.

## Data Availability

The data that support the findings of this study are available in Supporting Information of this article.
