# Supplementary material for: Evaluating zero‐shot prediction of monomeric protein design success by AlphaFold, ESMFold, and ProteinMPNN
Source: Protein Sci. 2026 Jan 20;35(2):e70453. doi: 10.1002/pro.70453 (PMC12817478; doi:10.1002/pro.70453)
Supplement: Supplementary file 2 — Figure S1. Most de novo designs failed experimentally due to not being monomeric. Counts of experimentally unsuccessful designs for each experimental criteria used to define design experimental success. Figure S2. Distribution of confidence metrics for experimental outcomes of designs. Distribution of confidence metrics comparing successful (blue) and unsuccessful designs (orange) across different experimental outcomes: expression, solubility, monomeric state, and secondary structure. Figure S3. Average AlphaFold2 pLDDT values are highly correlated with different recycle parameters. (a) Scatterplot capturing the relationship between AlphaFold2 average pLDDT for de novo designs computed using different recycle parameters. Green highlighted points indicate designs that increased their average pLDDT by >5 after increasing the recycles, and blue highlighted points are designs that increased their average pLDDT by >15 after increasing recycles. (b) AlphaFold2 predicted structures of designs that increased their average pLDDT >15 with increased recycles. (c) Boxplots showing the distribution of AlphaFold2 average pLDDT values using 25 recycles, comparing experimentally successful (blue) and unsuccessful (orange) designs. The ROC curves for AlphaFold2 average pLDDT (25 recycles) from bootstrapping on the whole dataset and individual article ROC curves. Figure S4. AlphaFold2 average PAE distributions for designed topologies. (a) Distributions of AlphaFold average PAE for designs included in this study. (b) Distribution of AlphaFold average PAE for experimentally successful designs (blue) and unsuccessful designs (orange) for each design topology. Figure S5. ESMFold average pLDDT distributions for designed topologies. (a) Distribution of ESMFold average pLDDT for designs included in this study. (b) Distribution of ESMFold average pLDDT for experimental successful designs (blue) and unsuccessful designs (orange) for each design topology Figure S6. ProteinMPNN score distributi [file PRO-35-e70453-s001.docx]

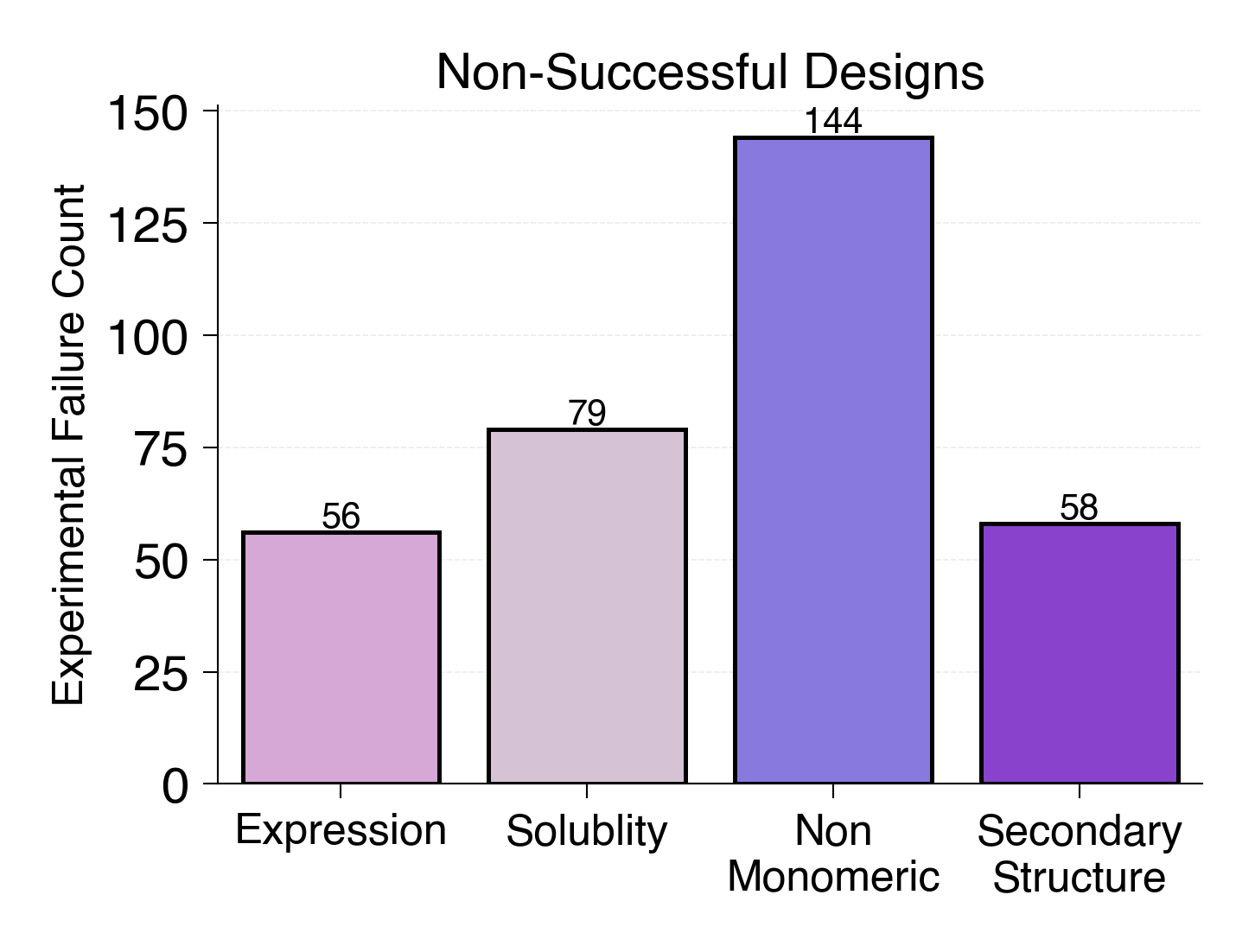


**Figure S1. Most *de novo* designs failed experimentally due to not being monomeric.** Counts of experimentally unsuccessful designs for each experimental criteria used to define design experimental success.


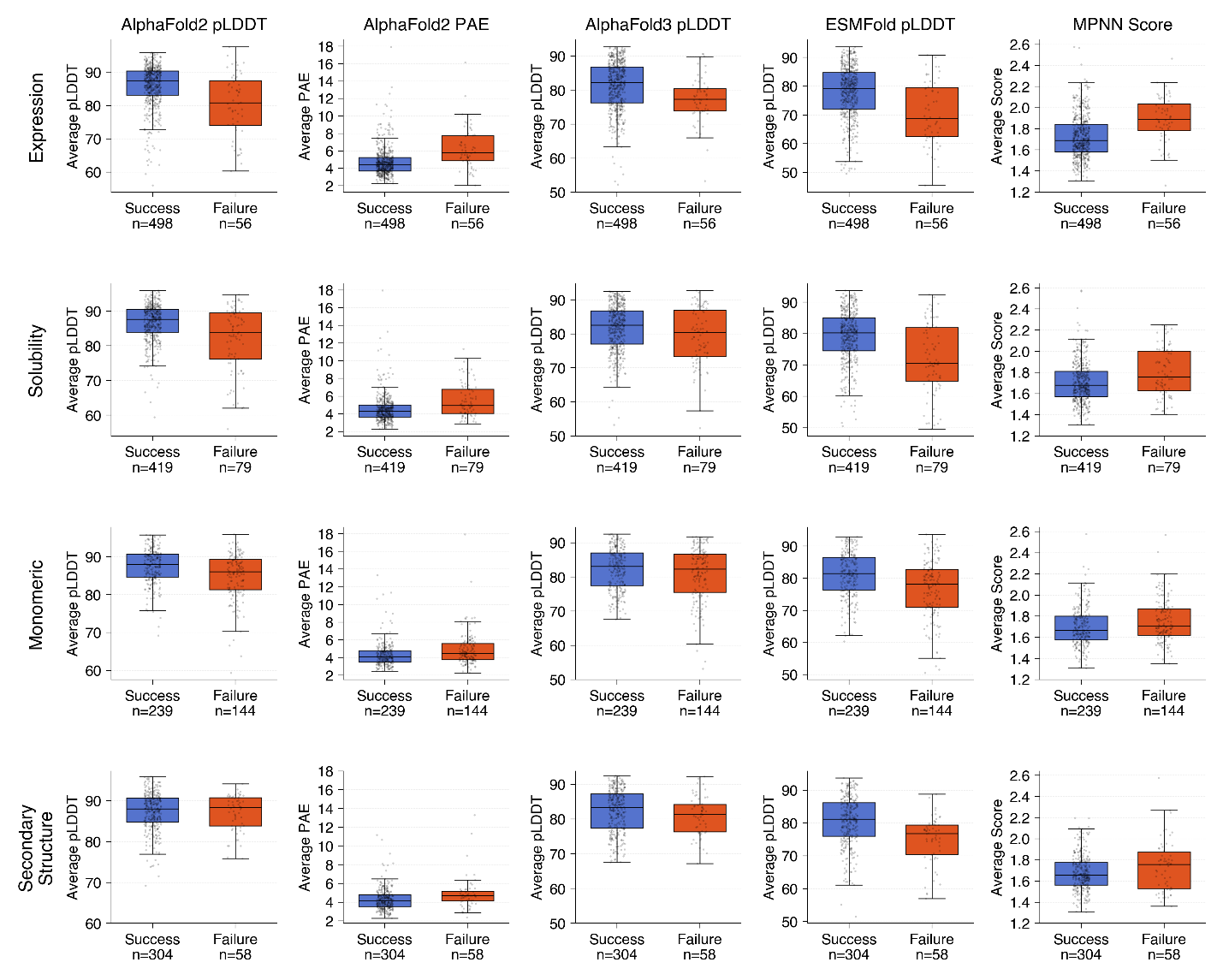


**Figure S2. Distribution of confidence metrics for experimental outcomes of designs.** Distribution of confidence metrics comparing successful (blue) and unsuccessful designs (orange) across different experimental outcomes: expression, solubility, monomeric state, and secondary structure.

**
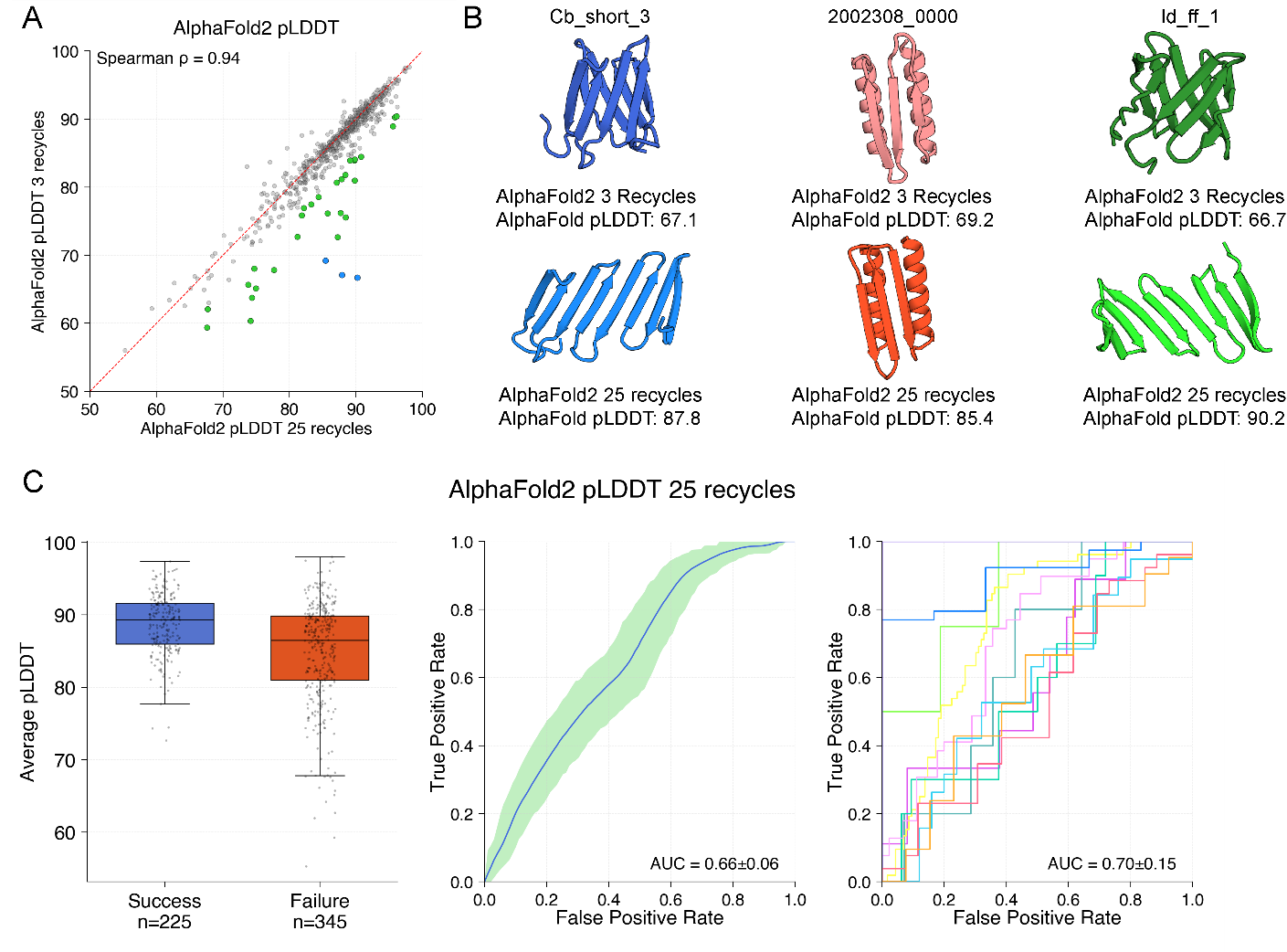
**

**Figure S3. Average AlphaFold2 pLDDT values are highly correlated with different recycle parameters.** **A)** Scatterplot capturing the relationship between AlphaFold2 average pLDDT for de novo designs computed using different recycle parameters. Green highlighted points indicate designs that increased their average pLDDT by >5 after increasing the recycles, and blue highlighted points are designs that increased their average pLDDT by >15 after increasing recycles. **B)** AlphaFold2 predicted structures of designs that increased their average pLDDT >15 with increased recycles. **C)** Boxplots showing the distribution of AlphaFold2 average pLDDT values using 25 recycles, comparing experimentally successful (blue) and unsuccessful (orange) designs. The ROC curves for AlphaFold2 average pLDDT (25 recycles) from bootstrapping on the whole dataset and individual article ROC curves.

**
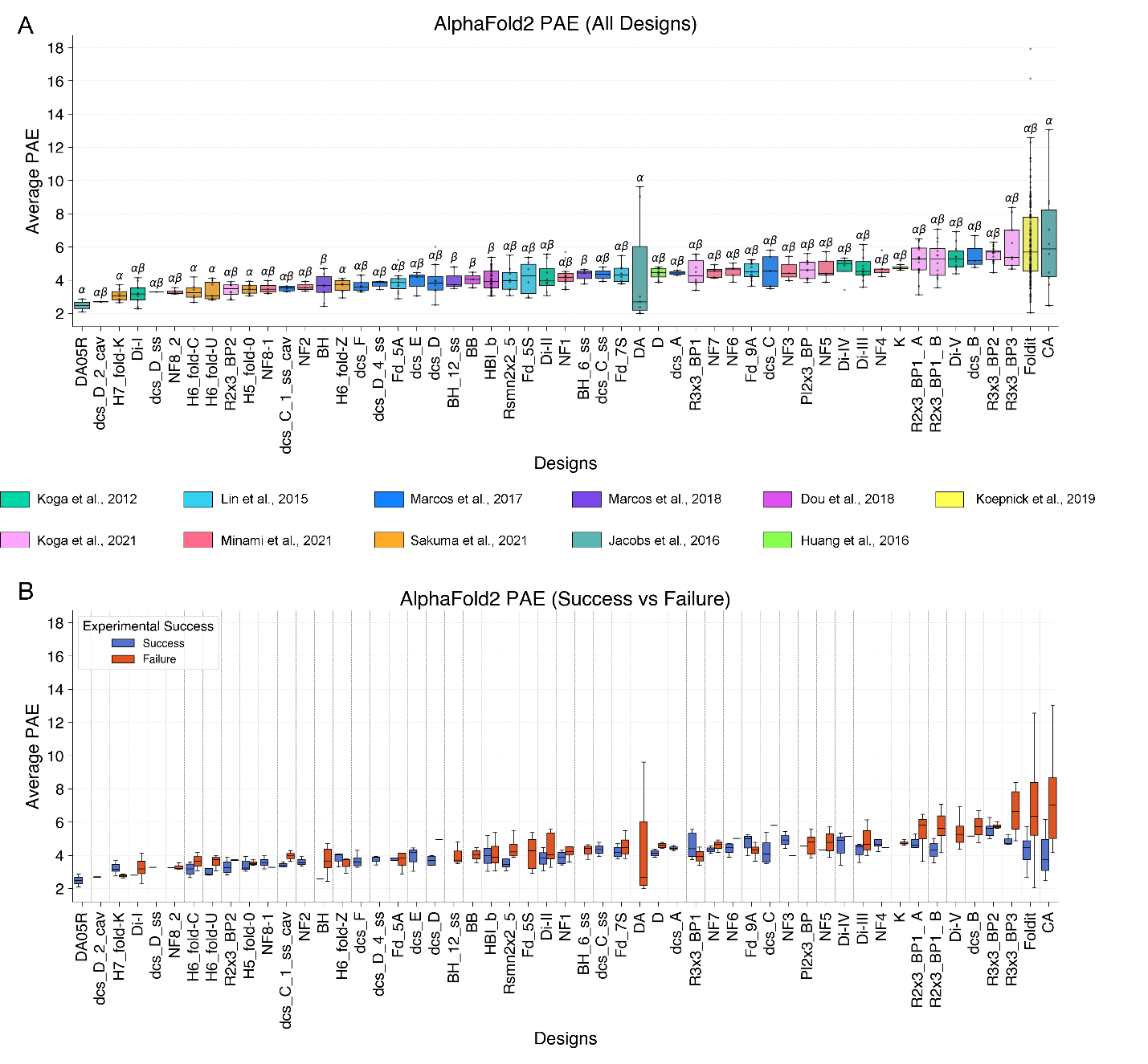
**

**Figure S4. AlphaFold2 average PAE distributions for designed topologies. A)** Distributions of AlphaFold average PAE for designs included in this study. **B)** Distribution of AlphaFold average PAE for experimentally successful designs (blue) and unsuccessful designs (orange) for each design topology.


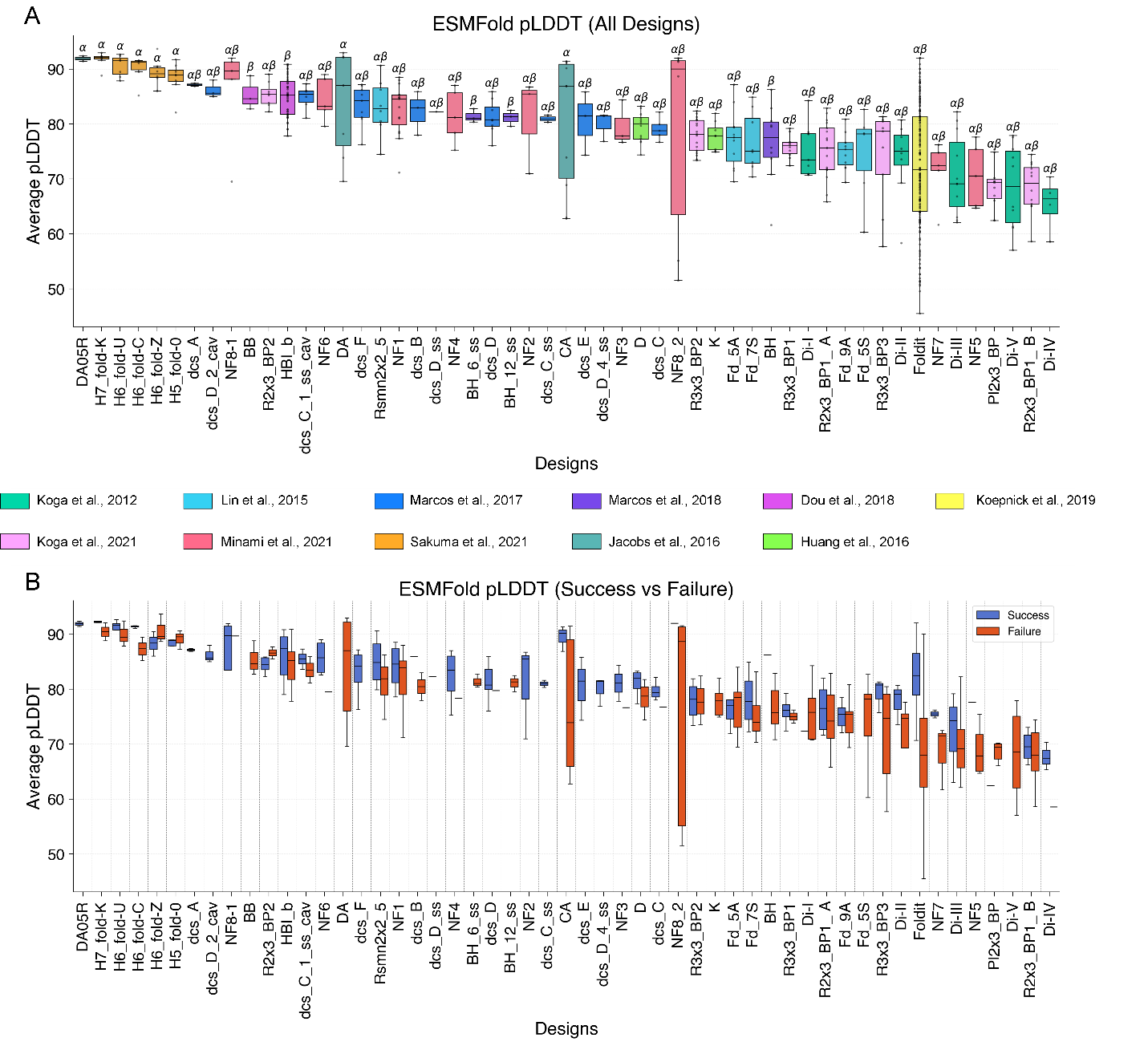


**Figure S5. ESMFold average pLDDT distributions for designed topologies. A)** Distribution of ESMFold average pLDDT for designs included in this study. **B)** Distribution of ESMFold average pLDDT for experimental successful designs (blue) and unsuccessful designs (orange) for each design topology


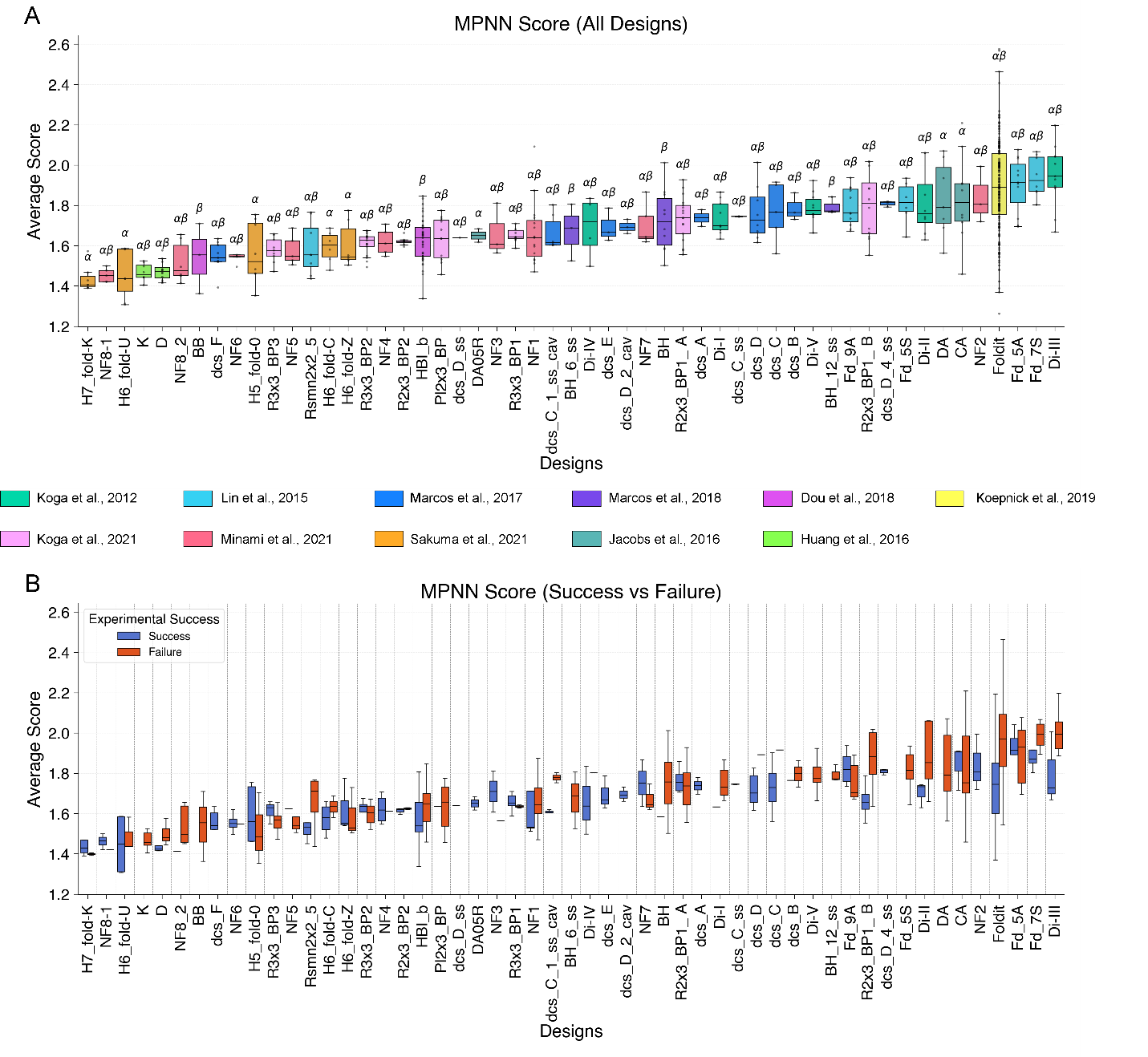


**Figure S6. ProteinMPNN score distributions for designed topologies. A)** Distribution of MPNN scores for designs included in this study. **B)** Distribution of MPNN score for experimental successful (blue) and unsuccessful designs (orange) for each design topology.


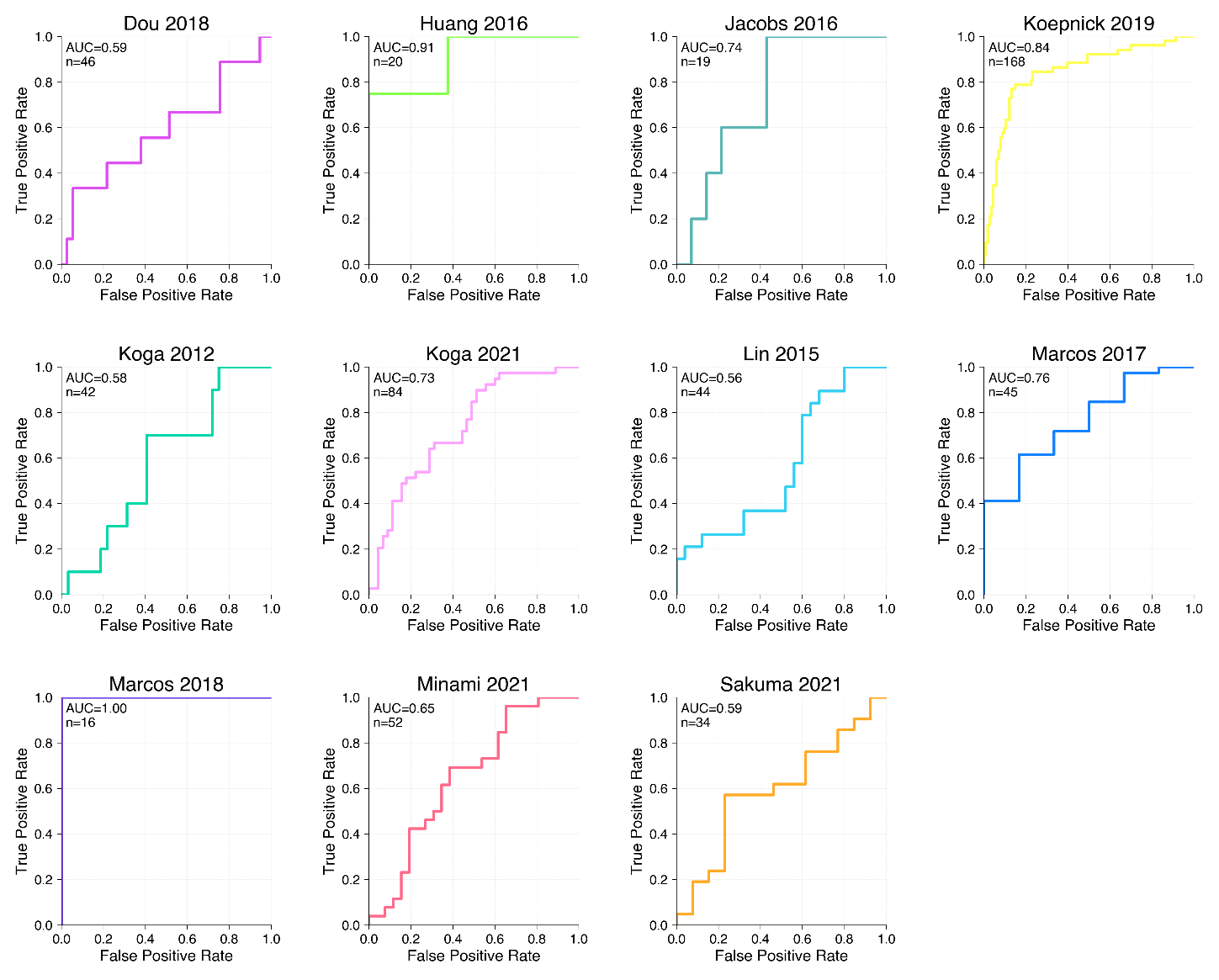


**Figure S7. Logistic regression models combining all confidence metrics to predict held-out designs from each study.** ROC curves were generated for each held-out study. The AUC and number of examples (n) are annotated in each plot.


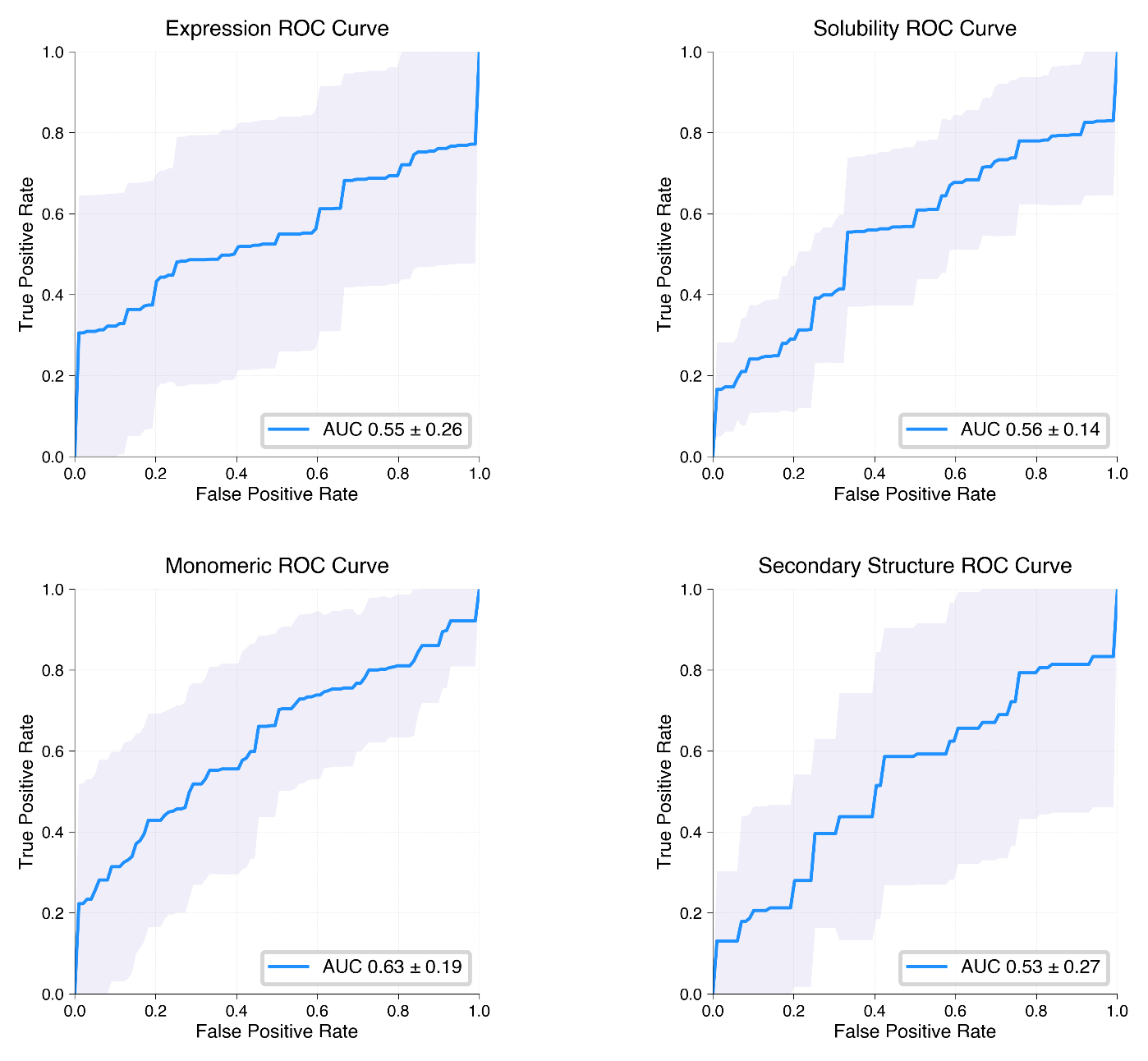


**Figure S8. Logistic regression models using all confidence metrics trained to predict whether designs were expressed, soluble, monomeric, and folded into designed secondary structure.** The ROC curves display the mean (dark blue) and standard deviation across held out test articles.


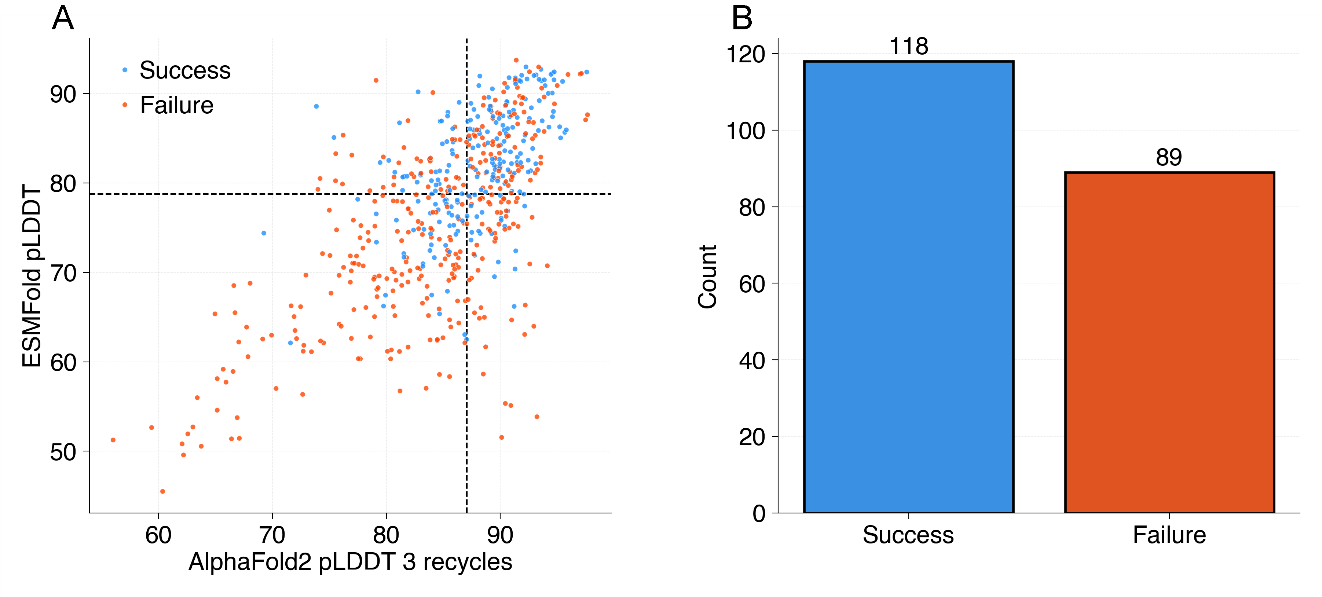


**Figure S9. Selecting the top 50% of designs according to AlphaFold2 pLDDT and ESMFold pLDDT increases design success rate to 57%. A)** Scatterplot displaying AlphaFold2 pLDDT and ESMFold pLDDT for all designs. Designs are labeled as experimentally successful (blue) or experimentally unsuccessful (orange). The dashed lines indicate the 50^th^ percentile for each metric and the top-right quadrant shows the top 50% of designs for both metrics. **B)** Counts of experimentally successful and unsuccessful designs for the top 50% designs.

**Explanation of Dataset**

**Name:** Design name

**Sequence:** Design protein sequence

**Sequence Length:** Design sequence length

**Expressed:** Design expression in *E. coli*

**Soluble:** Design solubility

**CD:** Secondary structure assessment monitored by circular dichroism

**Monomeric:** Assessing whether designs were monomeric according to size exclusion chromatography

**NMR:** Designs displaying well-resolved HSQC

**Fold:** Represents the design fold
Di-I - Ferredoxin-like fold

Di-II – Rossmann 2×2 fold

Di-III - IF3-like fold

Di-IV - P-loop 2×2 fold

Di-V – Rossmann 3×1 fold

Fd_5S - Ferredoxin-like fold containing 58 residues and a register shift between the first and third strands.

Fd_5A - Ferredoxin-like fold containing 66 residues, without register shift.

Fd_7S - Ferredoxin-like fold containing 74 residues and a register shift.

Fd_7A - Ferredoxin-like fold containing 76 residues, without a register shift.

Fd_9A - Ferredoxin-like fold containing 98 residues, without a register shift.

Rsmn2×2_5 - Rossmann Fold with 87 residues.

Rsmn2×2_6 - Rossmann Fold with 99 residues.

D - TIM Barrel

Dcs - Design Folds with curved β-sheets

BH - Jellyroll Fold

BB - β-barrel designs

DA and CA – Structurally diverse α-helical proteins

Pl2×3_BP - P-loop 2×3-fold

R2x3_BP1 – Rossmann 2×3-fold

R3×3_BP1 and R3x3_BP2 – Rossmann 3×3-fold

NF-8 - Novel αβ-fold structures (four-stranded β-sheet topologies)

H1–7 - Complicated all α-helical proteins were 1-7 represent the number of helices

FoldIt - 20 diverse folds designed by citizen scientists

**article:** Design article

**PDB:** Design contains structure deposited in the PDB

**Experimental Success:** Design was considered experimentally successful if it was expressed, soluble, monomeric, and had a CD spectrum consistent with the intended design fold as reported in each study

**Predicted Success:** Classification predictions of experimental success from logistic regression model performed using combined confidence metrics

**Predicted Probability:** Probability estimated from logistic regression model that a design will be experimentally successful. The probabilities were used to generate ROC curve by ranking predictions across all thresholds.

**AlphaFold2 pLDDT 3 recycles:** Average AlphaFold2 pLDDT with 3 recycles

**AlphaFold pLDDT 25 recycles:** Average AlphaFold2 pLDDT with 25 recycles

**ESMFold pLDDT:** Average ESMFold pLDDT

**MPNN Score:** MPNN design score

**AlphaFold2 PAE:** Average AlphaFold2 PAE

**AlphaFold3 pLDDT:** Average AlphaFold3 pLDDT
